# Fast-Charging Sodium-Ion Batteries Enabled by Molecular-Level Designed Nitrogen and Phosphorus Codoped Mesoporous Soft Carbon

**DOI:** 10.34133/research.0209

**Published:** 2023-08-16

**Authors:** Lei Liu, Zhuzhu Du, Jiaqi Wang, Hongfang Du, Sheng Wu, Mengjun Li, Yixuan Zhang, Jinmeng Sun, Zhipeng Sun, Wei Ai

**Affiliations:** ^1^ Frontiers Science Center for Flexible Electronics (FSCFE) and Shaanxi Institute of Flexible Electronics (SIFE), Northwestern Polytechnical University (NPU), 127 West Youyi Road, Xi’an 710072, China.; ^2^ Fujian Cross Strait Institute of Flexible Electronics (Future Technologies), Fujian Normal University, Fuzhou 350117, China.; ^3^ School of Materials and Energy, Guangdong University of Technology, Guangzhou, 510006 Guangdong, China.

## Abstract

Soft carbons have attracted extensive interests as competitive anodes for fast-charging sodium-ion batteries (SIBs); however, the high-rate performance is still restricted by their large ion migration barriers and sluggish reaction kinetics. Herein, we show a molecular design approach toward the fabrication of nitrogen and phosphorus codoped mesoporous soft carbon (NPSC). The key to this strategy lies in the chemical cross-linking reaction between polyphosphoric acid and p-phenylenediamine, associated with pyrolysis induced in-situ self-activation that creates mesoporous structures and rich heteroatoms within the carbon matrix. Thanks to the enlarged interlayer spacing, reduced ion diffusion length, and plentiful active sites, the obtained NPSC delivers a superb rate capacity of 215 mAh g^−1^ at 10 A g^−1^ and an ultralong cycle life of 4,700 cycles at 5 A g^−1^. Remarkably, the full cell shows 99% capacity retention during 100 continuous cycles, and maximum energy and power densities of 191 Wh kg^−1^ and 9.2 kW kg^−1^, respectively. We believe that such a synthetic protocol could pave a novel venue to develop soft carbons with unique properties for advanced SIBs.

## Introduction

Fast-charging sodium-ion batteries (SIBs) are expected to break the limit of long charging times and accelerate the development of grid-scale storage [1,2]. So far, various anode materials (e.g., alloy, transition metal chalcogenides, and hard carbons) have been intensively investigated to accommodate more Na^+^ for fast-charging SIBs [3–5]. However, the large volume variations associated with sluggish Na^+^ (de)intercalation processes severely hinder their fast-charging performance. Soft carbons, featuring high flexibility and good rigidity, are capable of buffering the huge volume expansion and maintaining the structural stability [6]. In particular, their tunable crystallinity and lattice spacing can ensure rapid ion diffusion to well satisfy the dramatically increased external electrons at high current density [7]. Nevertheless, soft carbons usually express inferior rate performances because of the large ion migration barriers in their interior region [8]. Given that the diffusion time (*t*) of Na^+^ in the electrode materials can be calculated from the equation of *t* = *x*^2^/*qD*, where the *x*, *q*, and *D* are assigned to the diffusion path, dimensionality constant, and diffusion coefficient, respectively [9]. Therefore, the construction of porous structures is conducive to shorten the Na^+^ diffusion length (low *x*) and achieve a 3-dimensional diffusion model (high *q*), which could address the kinetic issues of soft carbons. Conventional methods for constructing porous structures in soft carbons normally involve chemical activation and/or hard template, which pose challenges in achieving a homogeneous nanostructure. Besides, their complex routes and harsh synthesis conditions also bringing some economic and environmental concerns [10–12]. To this end, developing facile and accurate approaches for constructing porous structure is crucial yet challenging for rapid Na^+^ storage in soft carbons.

Beyond designing porous structures, further structural engineering of soft carbons can also improve the Na^+^ diffusion coefficient (high *D*) and thereby dramatically boost the fast-charging capability [13–15]. Typically, doping heteroatoms (e.g., N, P, and S) into the carbon lattices has been demonstrated to be very efficient in regulating the structural and electronic properties of soft carbons, which would drastically accelerate their ion diffusion kinetics [16–18]. Currently, 2 main synthetic strategies have been widely applied to incorporate heteroatoms into soft carbons. One is the annealing of petroleum by-products (e.g., pitch and petroleum coke) with heteroatom-containing sources (e.g*.*, urea and triphenylphosphine) [6,8,19,20]. The other approach is the direct pyrolysis of thermoplastic polymers that possess heteroatom moieties [21,22]. However, the aromatic hydrocarbon molecules within these precursors tend to stacking because of the strong π-π interactions, leading to small interlayer distance and low heteroatom contents [23]. Such characteristics are unfavorable to the rapid Na^+^ storage and therefore result in limited improvement in rate performance of soft carbons. To further improve the Na^+^ transport capability, codoping with 2 different heteroatoms (e.g., N/P and N/S) is a promising option since the synergistic effects of the codopants could not only expand the interlayer spacing to reduce the ion migration barriers but also create more defect sites for Na^+^ storage [24–26]. Based on the above considerations, an effective approach is expected to prepare soft carbons with fascinating porous structure and high-level codopants to accelerate the Na^+^ diffusion kinetics.

Herein, a molecular design strategy is developed for the fabrication of mesoporous soft carbons that simultaneously incorporates N and P into the carbon framework. This strategy involves the pyrolysis of a polymer formed by the chemical cross-linking reaction between polyphosphoric acid (PPA) and p-phenylenediamine (PA), which could achieve in-situ self-activation to produce porous structures and facilitate uniform N/P doping. Such an impressive nanostructure dramatically enhances the ion diffusion kinetics, rendering excellent rate capability (215 mAh g^−1^ even at 10 A g^−1^) and long-term cycling stability (4,700 cycles). When coupled with Na_3_V_2_(PO_4_)_3_F_3_@C (NVPFC) cathode, the full cell exhibits a high energy density of 191 Wh kg^−1^, a maximum power density of 9.2 kW kg^−1^, and a good capacity retention of 99% after 100 cycles. The proposed molecular design strategy provides a new route for the preparation of advanced carbon materials with intricate nanostructures for high-performance ions storage.

## Results

**Fig. 1.**
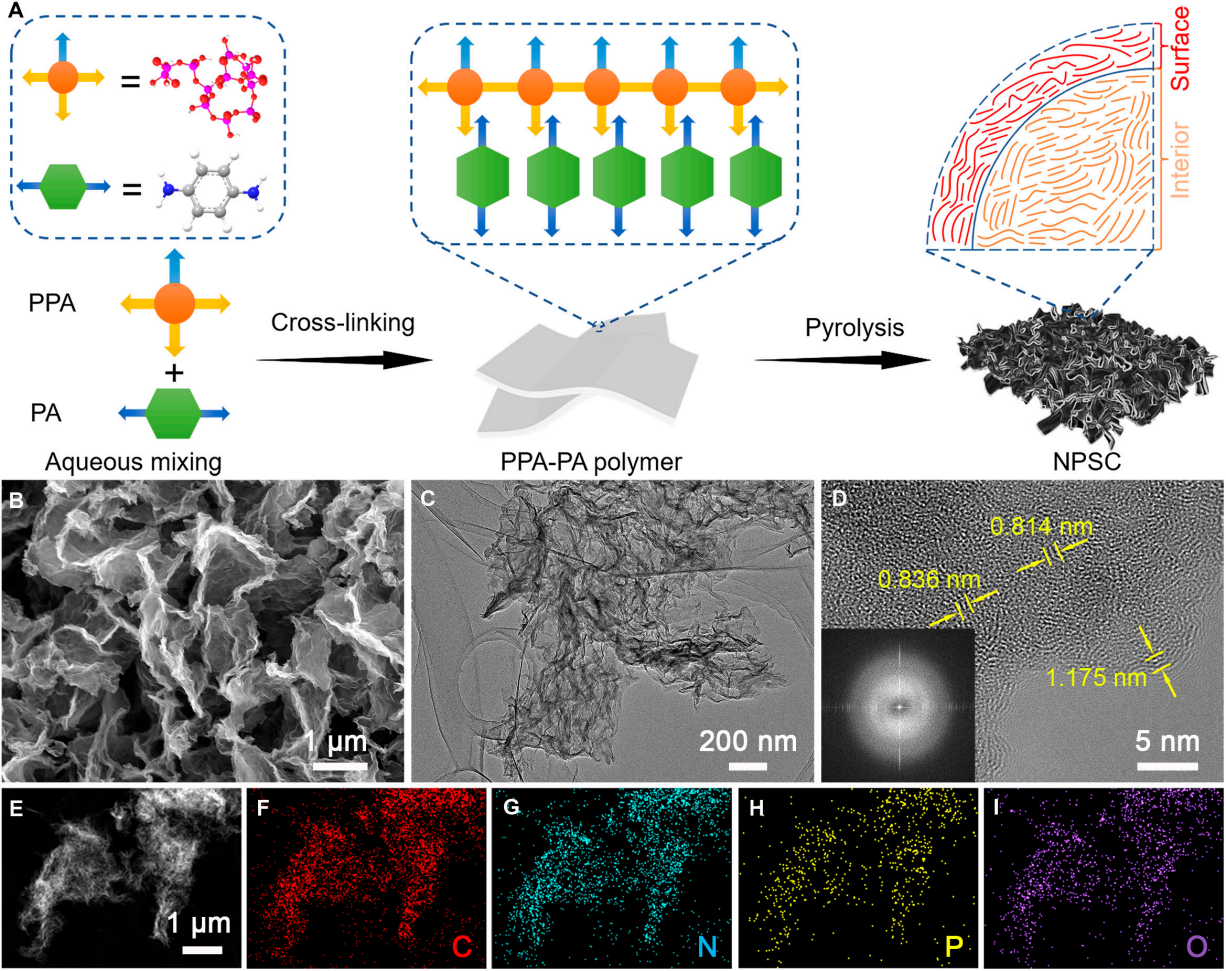
(A) Schematic illustration of the molecular design strategy for the fabrication of NPSC. The surface region is defined as extending from the surface to inner with a distance of 10 nm, while the area beyond 10 nm is considered as the internal region. (B) SEM, (C) TEM, (D) HRTEM along with the fast Fourier transform image, and (E to I) scanning transmission electron microscopy image and corresponding mappings of NPSC-800.

Figure 1A schematically illustrates the fabrication process of nitrogen and phosphorus codoped mesoporous soft carbon (NPSC), which involves a chemical cross-linking reaction between PPA and PA, associated with a subsequent pyrolysis process. Typically, PPA and PA first react to form PPA-PA through the strong electrostatic bonding, where PPA serves as a strong scaffold enabling the polymer to be highly thermostable (Fig. S1) [27]. At temperature above 750 °C, PPA-PA are carbonized accompanied by the production of polycyclic aromatic hydrocarbons and active phosphorus species [28]. The phosphorus species not only retard the directional arrangement of aromatic skeletons but also can afford in-situ self-activation to generate porous structures in the carbon products. Considering that the phosphorus species can participate in the self-activation process from the inside out, the resulting NPSC materials (denoted as NPSC-T, where T is the pyrolysis temperature) display a homogeneous nanostructure in the bulk state. According to the scanning electron microscopy (SEM) image, the as-prepared NPSC-800 exhibits a sheet-like morphology with carbon nanosheets cross-linked to form an open porous structure (Fig. 1B). In addition, there are abundant wrinkles attached to the surface of carbon skeleton, which could be readily observed by a transmission electron microscopy (TEM) image (Fig. 1C). These features would efficiently shorten the ion diffusion length and facilitate electrolyte penetration into the inner region, which is advantageous for rapid Na^+^ storage. High-resolution TEM (HRTEM) image of NPSC-800 shows a turbostratically amorphous structure, which is consistent with the corresponding fast Fourier transform image of selected-area electron diffraction (Fig. 1D). Notably, some short turbostratic graphitic nanodomains are distributed in the interior and surface regions with an average interlayer spacing ranging from 0.391 to 0.418 nm. Scanning transmission electron microscopy image of NPSC-800 and the corresponding elemental mappings demonstrated that the N, P, and O species are uniformly distributed on the carbon framework (Fig. 1E to I).

**Fig. 2.**
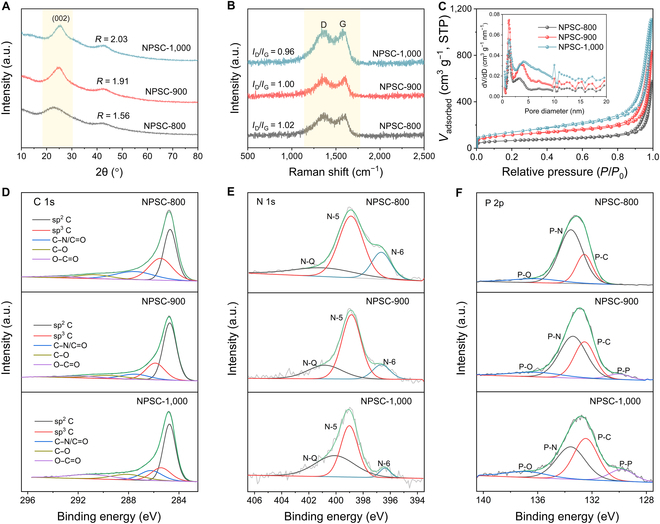
(A) XRD patterns, (B) Raman spectra, and (C) N_2_ adsorption–desorption isotherms of NPSC-800, NPSC-900, and NPSC-1,000. The inset of (C) shows the pore size distribution plot of the corresponding sample. High-resolution (D) C 1s, (E) N 1s, and (F) P 2p XPS spectra of NPSC-800, NPSC-900, and NPSC-1,000. a.u., arbitrary units.

With increasing the pyrolysis temperature, NPSC-900 and NPSC-1,000 also show similar morphology to NPSC-800 (Fig. S2), but their microstructures are different. X-ray diffraction (XRD) patterns of the NPSC samples are shown in Fig. 2A, where the 2 broad and dispersive diffraction peaks at about 24° and 44°can be well indexed to the (002) and (100) planes of carbon, respectively. The average interlayer spacings are calculated to be 0.389 nm for NPSC-800, 0.358 nm for NPSC-900, and 0.351 nm for NPSC-1,000. Their crystallinity degree was further characterized by the representative parameter *R* value (Fig. S3) [29]. The *R* value for NPSC-800 is 1.56, which increases to 1.91 for NPSC-900 and 2.03 for NPSC-1,000, demonstrating the increased graphitization degree in these samples. When the pyrolysis temperature is increased to 2,100 °C, the as-prepared NPSC-2,100 sample shows obvious diffraction peaks (26°, 43°, and 53°) resembling graphite (Fig. S4), verifying that the NPSC materials exhibit characteristics of soft carbon. Raman spectra present 2 distinct peaks at around 1,360 and 1,590 cm^−1^, which are associated to the disorder-induced D-band and the in-plane vibrational G-band, respectively. The *I*_D_/*I*_G_ (where *I*_D_ and *I*_G_ represent the Raman intensity of the D-band and G-band, respectively) for NPSC-800 is higher than that of NPSC-900 and NPSC-1,000, indicating its lower graphitization degree (Fig. 2B) [30], which is consistent with the HRTEM and XRD results. The specific surface areas and pore size distributions of the NPSC samples were evaluated by N_2_ adsorption–desorption isotherms (Fig. 2C). All the samples show typical type-IV isotherms, and the calculated Brunauer–Emmett–Teller specific surface areas of NPSC-800, NPSC-900, and NPSC-1,000 are 222, 397, and 492 m^2^ g^−1^, respectively. The increase of specific surface areas upon increasing the pyrolysis temperature could be attributed to the intensified reaction process with releasing more volatile gases [31]. Pore size distribution curves state that all the NPSC samples exhibit predominant mesopore structures with *V*_meso_ contributing more than 95% of the total pore volume (Table S1). Note that mesoporous structures are advantageous for fast ion transport and sufficient electrolyte accessibility for the charge transfer process of electrochemical reactions.

X-ray photoelectron spectroscopy (XPS) survey spectra confirm the existence of C, N, P, and O in NPSC samples (Fig. S5 and Table S2). The high-resolution C 1s XPS spectra display 5 peaks at 284.7, 285.9, 286.7, 288.2, and 290.7 eV, successively corresponding to the sp^2^ C (basal-plane), sp^3^ C (defect carbon), C–N/C=O, C–O, and O–C=O groups (Fig. 2D) [27,32,33]. The proportions of sp^3^/sp^2^ gradually decreased with the increase of pyrolysis temperature, confirming that NPSC-800 has a higher ratio of structural defects than NPSC-900 and NPSC-1,000 (Fig. S6). The N 1s spectra can be deconvoluted into 3 different peaks, assignable to the quaternary N (N-Q, ~400.9 eV), pyrrolic N (N-5, ~398.9 eV), and pyridinic N (N-6, ~396.7 eV) (Fig. 2E) [17,34]. Clearly, the edge-N (N-5 and N-6) ratio in NPSC-800 reaches 79%, while this value decreases to 75.6% for NPSC-900 and 54.1% for NPSC-1,000 (Fig. S7 and Table S3). Accordingly, the edge-N doping level in NPSC-800 is determined to be as high as 5.3 at%. The P 2p spectra further reveal the existence of P–O (~136.2 eV), P–N (~133.4 eV), and P–C (~122.5 eV) bonds in NPSC samples (Fig. 2F) [35]. The prevalence of the P–N bond configuration indicates that P dopants are prone to substitute C atoms and then bond with N atoms to form a N-rich structure [36]. The high content of P–N bonds (65.7%) in NPSC-800 could induce abundant structural defects and edge sites by distorting the integrated carbon plane (Fig. S8 and Table S4). In addition, the proportion of P–C bonds in NPSC-900 and NPSC-1,000 gradually increases due to the decomposition of edge-N configurations or the formation of in-plane N-Q at higher annealing temperature. Furthermore, P–P bonds (~130.1 eV) are formed between adjacent P-rich carbon layers, resulting in limited expansion of the carbon layers [37]. Together with the mesoporous structure and high N/P doping level, NPSC-800 is expected to display high Na^+^ storage capacity and excellent rate performance.

**Fig. 3.**
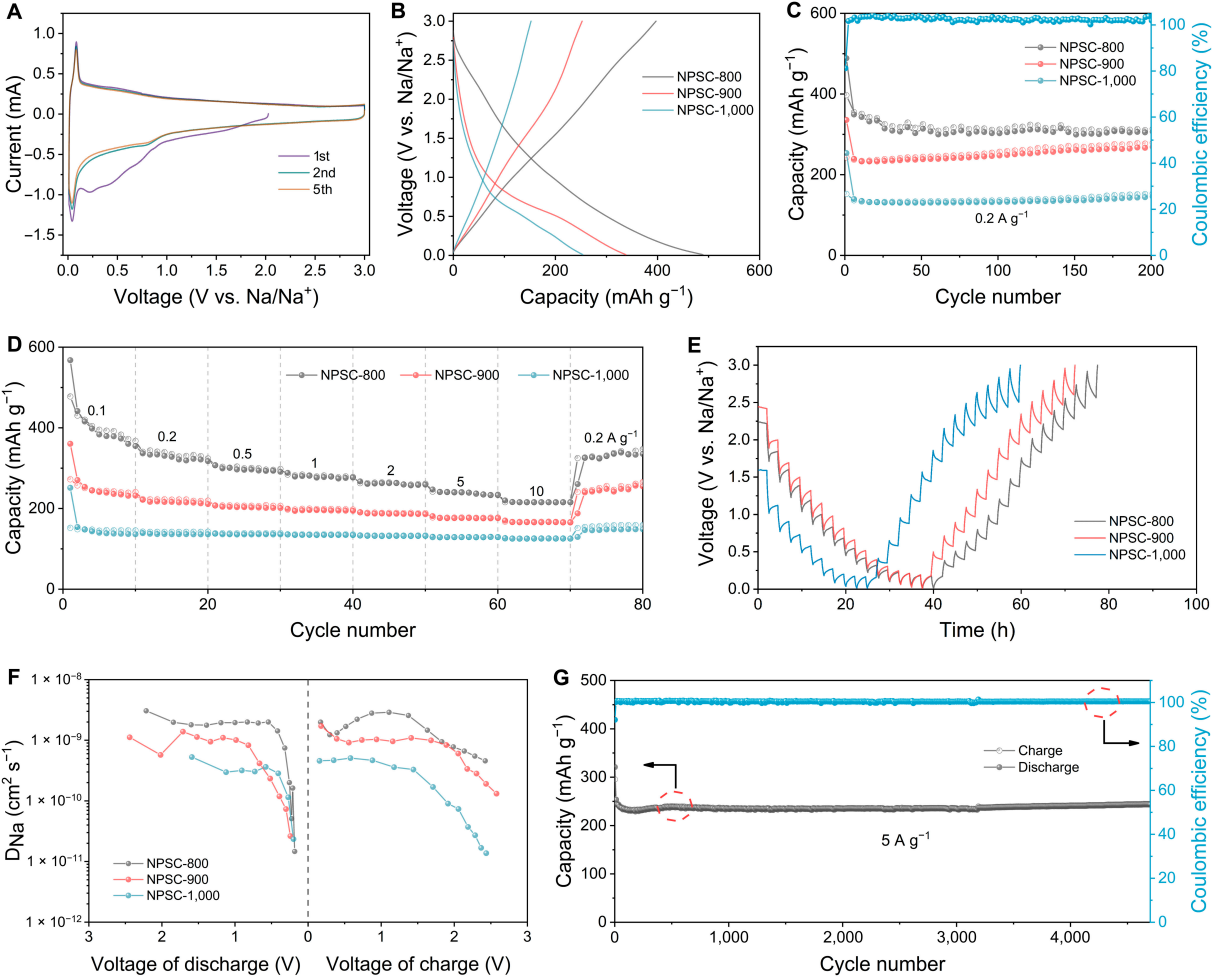
(A) CV curves of NPSC-800 at 0.5 mV s^−1^. (B) GCD profiles and (C) cycling performance of NPSC samples at 0.2 A g^−1^. (D) Rate capability, (E) galvanostatic intermittent titration technique potential profiles, and (F) the corresponding *D*_Na_ of NPSC samples. (G) Long-term cycling stability of NPSC-800 at 5 A g^−1^.

The Na^+^ storage properties of NPSC samples were first investigated by cyclic voltammetry (CV), where the broad reduction peak ranging from 0.2 to 0.8 V in the first cathodic scan can be assigned to the formation of solid electrolyte interphase film (Fig. 3A and Fig. S9). The sharp redox peaks centered at 0.1 V are associated with the (de)intercalation of Na^+^ into the graphitic nanodomains. In the following cycle, the cathodic peak at 0.8 V is ascribed to the redox reactions between N dopants and Na^+^. The almost overlap of the second to fifth CV curves indicates the highly reversible electrochemical behavior. Figure 3B shows the initial galvanostatic charge/discharge (GCD) profiles of NPSC samples at 0.2 A g^−1^, which exhibit typical sloping curves belonging to the surface-controlled capacitive process. The NPSC-800 exhibit an initial discharge capacity of 489 mAh g^−1^, while the charge capacity is 397 mAh g^−1^. The corresponding initial Coulombic efficiency is 81.2%, which can be ascribed to the formation of a uniform solid electrolyte interphase layer that functions as a physical barrier to block further side reactions (Fig. S10) [38,39]. Despite similar Na^+^ storage behavior, a decreased initial Coulombic efficiency was observed in NPSC-900 (74.5%) and NPSC-1,000 (60.4%), which is related to their larger surface areas that accelerate the electrolyte decomposition. After 200 continuous cycles at 0.2 A g^−1^, the NPSC-800 displays a high capacity of 314 mAh g^−1^ (Fig. 3C), higher than that of NPSC-900 (268 mAh g^−1^) and NPSC-1,000 (152 mAh g^−1^). The remarkable Na^+^ storage properties of NPSC-800 are subsequently verified by its superior rate capability (Fig. 3D). Compared to NPSC-900 and NPSC-1,000, the NPSC-800 electrode shows consistently higher reversible capacities at different current densities. Additionally, when the current density increases to 10 A g^−1^, NPSC-800 can still deliver a reversible capacity of 215 mAh g^−1^. As shown in Fig. S11, the rate capability of NPSC-800 is superior to most of the well-designed carbon anodes in the literature. More importantly, the specific capacity of NPSC-800 reaches up to 132 mAh g^−1^ at 10 A g^−1^ at the voltage below 1 V (Fig. S12), implying its great potential for fast-charging SIBs. The outstanding electrochemical performances of NPSC-800 are related to the synergy of mesoporous structures and rich dopants, which markedly promote ion diffusion and electron transfer. As confirmed by the electrochemical impedance spectroscopy shown in Fig. S13 and Table S5, NPSC-800 exhibits a charge transfer resistance (*R*_ct_) of only 5.2 Ω, dramatically lower than NPSC-900 (15.7 Ω) and NPSC-1,000 (36.5 Ω). Further galvanostatic intermittent titration techniqueresults also verify that NPSC-800 has a lower overpotential and higher Na^+^ diffusion coefficient (*D*_Na_) than NPSC-900 and NPSC-1,000 (Fig. 3E and F). Benefiting from these advantages, NPSC-800 delivers high-rate cycling stability at 5 A g^−1^, with a high capacity of 244 mAh g^−1^ after 4,700 cycles (Fig. 3G). Such a remarkable fast-charging capability far exceeds the previously reported carbon anodes (Table S6).

**Fig. 4.**
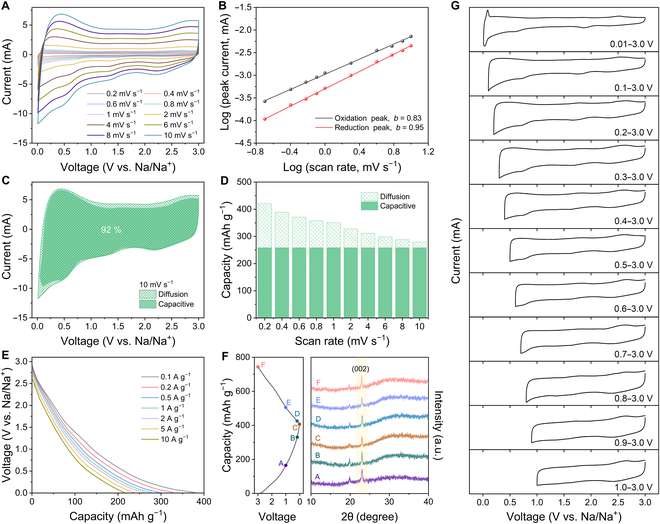
Electrochemical tests of NPSC-800. (A) CV curves from 0.2 to 10 mV s^−1^ and (B) the calculation of *b* values at the corresponding redox peaks. (C) Separation of the capacitive- and diffusion-controlled contributions at 10 mV s^−1^. (D) The capacities contributed by the capacitive and diffusion processes under different scan rates. (E) Discharge profiles at various current densities. (F) The ex-situ XRD patterns. (G) CV curves under different cutoff voltages at 0.5 mV s^−1^.

To clarify the real origin of good rate capability of NPSC-800, we performed CV analyses at various scan rates. As can be observed, the CV curves possess similar profiles along with the increasing scan rate (Fig. 4A), reflecting the fast ion diffusion kinetics and minor polarization of NPSC-800 at high rates. The scan rate (*v*) and peak current (*i*) obey Eq. 1 [40]:

$$i=av^{b}$$ (1)

where *a* and *b* are adjustable parameters. When the *b* value gets close to 0.5, the electrochemical reaction follows a diffusion-controlled intercalation process, while approaching 1 indicates a surface-induced capacitive process.

Fig. 4B displays the plots of NPSC-800 for the oxidation and reduction peaks, where the *b* values are calculated to be 0.83 and 0.95, respectively, reflecting a favorable capacitive-controlled process for rapid Na^+^ storage. According to Eq. 2 [41]:

$$i=k_{1}v+k_{2}v^{1/2}$$ (2)

the capacitive-controlled (*k*_1_*v*) and diffusion-controlled (*k*_2_*v*^1/2^) contributions to the overall capacity can be obtained. As shown in Fig. 4C and Fig. S14, the capacitive contribution gradually grows with increasing scan rates and finally reaches 92% at 10 mV s^−1^. Intriguingly, the capacitive capacity presents a constant value of around 258 mAh g^−1^ under progressively increased scan rates. By contrast, the diffusive capacity decreases along with the increases of scan rate from 0.2 to 10 mV s^−1^ (Fig. 4D) [42]. Moreover, the discharge profiles of NPSC-800 at different current densities possess an approximately linear line, further confirming its capacitive behavior for high-power operation (Fig. 4E). Ex-situ XRD at different discharge/charge states were carried out to evaluate the Na^+^ storage behavior of NPSC-800 (Fig. 4F). The position of (002) peak shows negligible change, indicating that the excellent Na^+^ storage properties are mainly governed by the adsorption process in NPSC-800. In addition, we further explored the in-depth reaction mechanism based on the CV curves with different voltage windows. As shown in Fig. 4G, the CV curves present a rectangular shape at the voltage range of 1.0 to 3.0 V, which can be attributed to the capacitive behavior of Na^+^ adsorption on the intrinsic defects of carbon. With the cutoff voltage decrease to 0.1 V, the CV profiles gradually show an obvious tailing phenomenon, indicating that NPSC-800 has undergone a rapid faradic reaction caused by the incorporation of Na^+^ at the doping sites. In addition, the intercalation of Na^+^ in the graphitic domains also induces a distinct polarization of the CV at the cutoff voltage near 0.01 V.

**Fig. 5.**
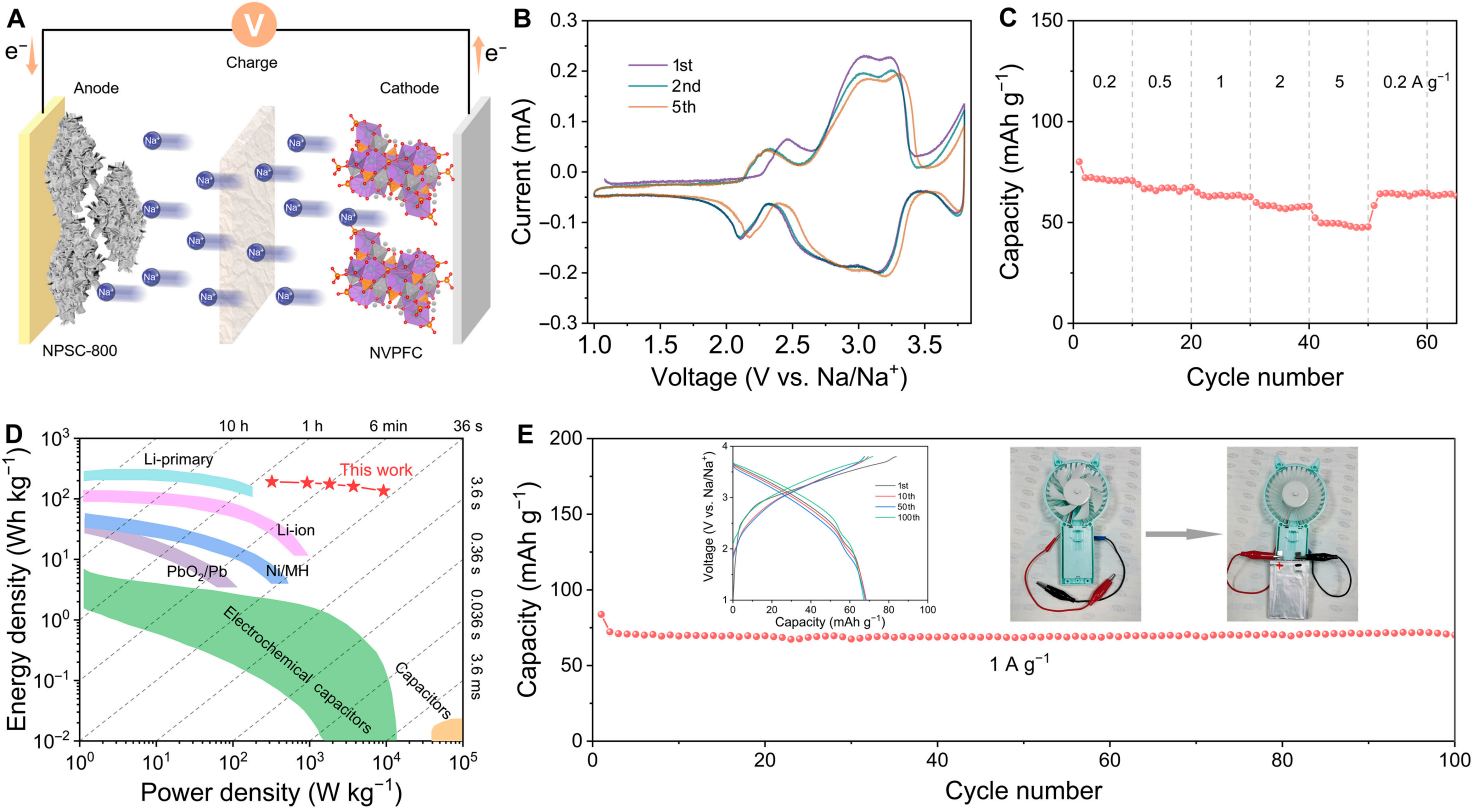
Electrochemical tests of NPSC-800//NVPFC full cell. (A) Schematic diagram of the battery configuration. (B) CV curves at 0.5 mV s^−1^. (C) Rate performance at different current densities. (D) Ragone plot. (E) Cycling stability at 1 A g^−1^. The inset is the corresponding GCD profiles and the digital photographs of an electric fan powered by an assembled pouch-type full cell.

Full cells were further assembled using presodiated NPSC-800 as anode and NVPFC as cathode (Fig. 5A). The active mass of the electrodes is tuned to make the negative-to-positive capacity ratio around 1.2 (Fig. S15), and the full cells were cycled in a potential range of 1.0 to 3.8 V to avoid electrolyte decomposition during cycling. Both CV curves and GCD profiles of the NPSC-800//NVPFC full cell show obvious redox peaks (Fig. 5B and Fig. S16), reflecting the combined electrochemical processes of NPSC-800 and NVPFC. As shown in Fig. 5C, the full cell displays a decent reversible capacity of 80, 67, 64, and 58 mAh g^−1^ (calculated based on the total active mass of anode and cathode) at the current densities of 0.2, 0.5, 1, and 2 A g^−1^, respectively. Even at a fast-charging rate of 5 A g^−1^, corresponding to a charging time of 50 s, the full cell still deliver a high capacity of 50 mAh g^−1^. Ragone plot shows that the energy/power densities of our full cell are comparable and even better than the most representative energy storage devices reported in the literatures (Fig. 5D). Accordingly, a maximum energy density of the full cell reaches up to 191 Wh kg^−1^ at 322 W kg^−1^ and still harvests 135 Wh kg^−1^ at a high-power output of 9.2 kW kg^−1^. Cycling tests at 1 A g^−1^ show that the full cell has a good cycling performance with capacity retention of 99% over 100 cycles (from the 2nd to the 100th). In addition, the GCD profile at the 100th cycle is very similar to the 1st cycle, indicating a low polarization of the cell during cycling. Remarkably, the assembled pouch cell successfully actuates a miniature fan (inset of Fig. 5F), demonstrating the potential application of NPSC-800 for fast-charging SIBs.

## Discussion

In summary, we have developed a molecular design strategy for the synthesis of NPSC by using a predefined polymer (PPA-PA). The successful preparation of NPSC is mainly arising from the in-situ self-activation of PPA-PA during annealing, which effectively restrains the aromatic skeleton restacking and simultaneously facilitates pore formation and N/P codoping. These features make NPSC a superior anode material for fast-charging SIBs. As expected, the NPSC can display a high rate capability of 215 mAh g^−1^ at 10 A g^−1^ and a remarkable cycle durability up to 4,700 cycles. Impressively, the full cell shows a stable cycling stability with 99% capacity retention for over 100 cycles and provides a high energy/power density of 191 Wh kg^−1^/9.2 kW kg^−1^. We believe that this synthetic strategy will be helpful for the design of carbonaceous materials for fast-charging energy storage systems.

## Materials and Methods

### Material synthesis

PA (10 g) was dispersed into 100 ml of deionized water in a glass bottle and kept at 80 °C for 30 min. Under vigorous stirring, 5 ml of PPA was then added to obtain a coffee-like suspension. After 2-h stirring, the as-prepared suspension was centrifuged and thoroughly washed several times with deionized water and dried at 100 °C to obtain PPA-PA. The NPSC samples were obtained by annealing PPA-PA at the predetermined temperature (800, 900, and 1,000 °C) under Ar atmosphere for 2 h.

### Characterizations

The crystalline structure of NPSC samples was identified by XRD with Cu Kα radiation (Bruker D8) and Raman spectroscopy (Horiba LabRAM Evolution spectrometer). The morphology and microstructure were characterized by SEM (Verios G4) and TEM (FEI Talos F200X TEM). N_2_ adsorption–desorption analyses were carried out on an ASAP2460 apparatus. The surface chemical state and the corresponding configuration was analyzed by XPS (Axis Supra XPS spectrometer with Al Kα x-ray). Fourier-transform infrared spectroscopy was done with a Bruker Tensor II instrument.

### Electrochemical measurements

The anodic electrodes were prepared by dispersing NPSC, super P, and carboxymethyl cellulose with a mass ratio of 8:1:1 in deionized water, after which the slurry was coated onto copper foil and dried overnight at 100 °C. The areal mass loading on NPSC electrodes is ~0.8 to 1.4 mg cm^−2^. The cathodic electrodes were prepared by mixing NVPFC, super P, and polyvinylidene fluoride with a mass ratio of 8:1:1 in N-methyl-2-pyrrolidone to form a slurry, which was casted on a carbon-coated aluminum foil and then dried overnight at 60 °C. The half cells were assembled in an argon-filled glovebox with Na metal sheet as the counter electrode, 1.0 M NaPF_6_ in dimethyl ether as the electrolyte, and glass microfiber filter (Whatman) as the separator. For full-cell assembly, the NPSC anode was precycled 5 times at 0.05 A g^−1^ in a half-cell configuration, and the areal mass loading of NVPFC cathodes is ~3 to 4 mg cm^−2^.

## Data Availability

All data needed to evaluate the conclusions in the paper are present in the paper and/or the Supplementary Materials.
