# Supplementary material for: Fast-Charging Sodium-Ion Batteries Enabled by Molecular-Level Designed Nitrogen and Phosphorus Codoped Mesoporous Soft Carbon
Source: Research (Wash D C). 2023 Aug 16;6:0209. doi: 10.34133/research.0209 (PMC10430870; doi:10.34133/research.0209)
Supplement: Supplementary 1 — Figs. S1 to S16 Tables S1 to S6 [file research.0209.f1.docx]

Supporting Information

Fast-Charging Sodium-Ion Batteries Enabled by Molecular-Level Designed Nitrogen and Phosphorus Co-doped Mesoporous Soft Carbon

Lei Liu^1†^, Zhuzhu Du^1†^, Jiaqi Wang^1^, Hongfang Du^1,2^*, Sheng Wu^3^, Mengjun Li^1^, Yixuan Zhang^1^, Jinmeng Sun^1^, Zhipeng Sun^3^*, and Wei Ai^1^*

^1^Frontiers Science Center for Flexible Electronics (FSCFE) & Shaanxi Institute of Flexible Electronics (SIFE), Northwestern Polytechnical University (NPU), 127 West Youyi Road, Xi’an 710072, China.

^2^Fujian Cross Strait Institute of Flexible Electronics (Future Technologies), Fujian Normal University, Fuzhou 350117, China.

^3^School of Materials and Energy, Guangdong University of Technology, Guangzhou, 510006, Guangdong, China.

*Address correspondence to: iamhfdu@nwpu.edu.cn (H.D.); zpsunxj@gdut.edu.cn (Z.S.); iamwai@nwpu.edu.cn (W.A.)

†These authors contributed equally to this work.

**1. Supplementary Figures and Tables**

**
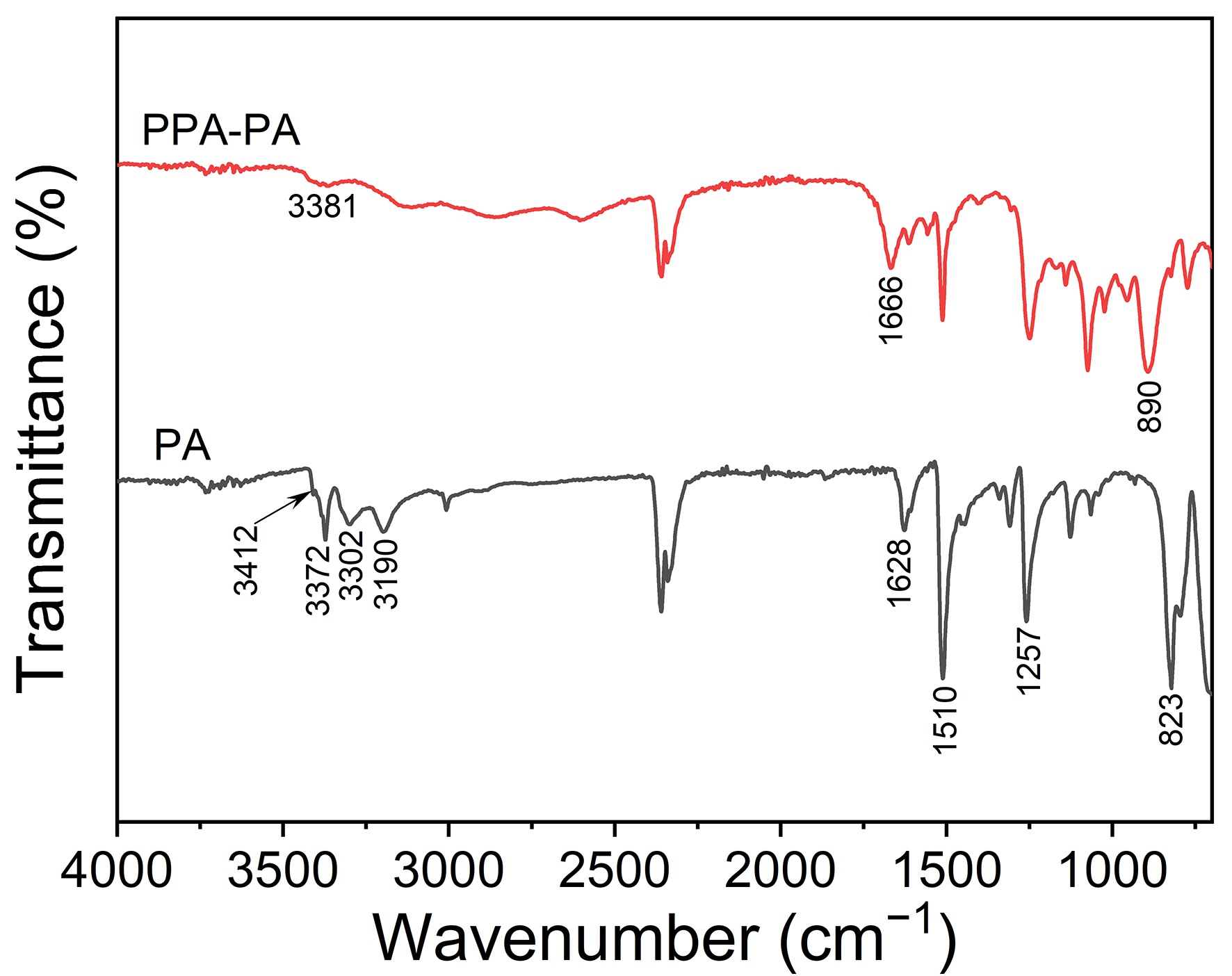
**

**Fig. S1.** FTIR spectra of PPA-PA polymer and PA.

The characteristic absorption peaks at around 3412 and 3372 cm^−1^ are assigned to the N-H stretches, whereas the peak at around 3302, 3190, and 1628 cm^−1^ are indexed to the N-H bending vibration. The other three representative peaks are assigned to the C=N stretching at around 1510 cm^−1^, C-N stretching at around 1257 cm^−1^, and N-H wag at around 823 cm^−1^.^1^ In contrast, the FTIR of PPA-PA display a red shift in the N-H peaks (∼1666 and 890 cm^−1^), and the characteristic N-H stretching band (∼3381 cm^−1^) almost disappears, indicating the successful condensation reaction between PPA and PA.^2, 3^


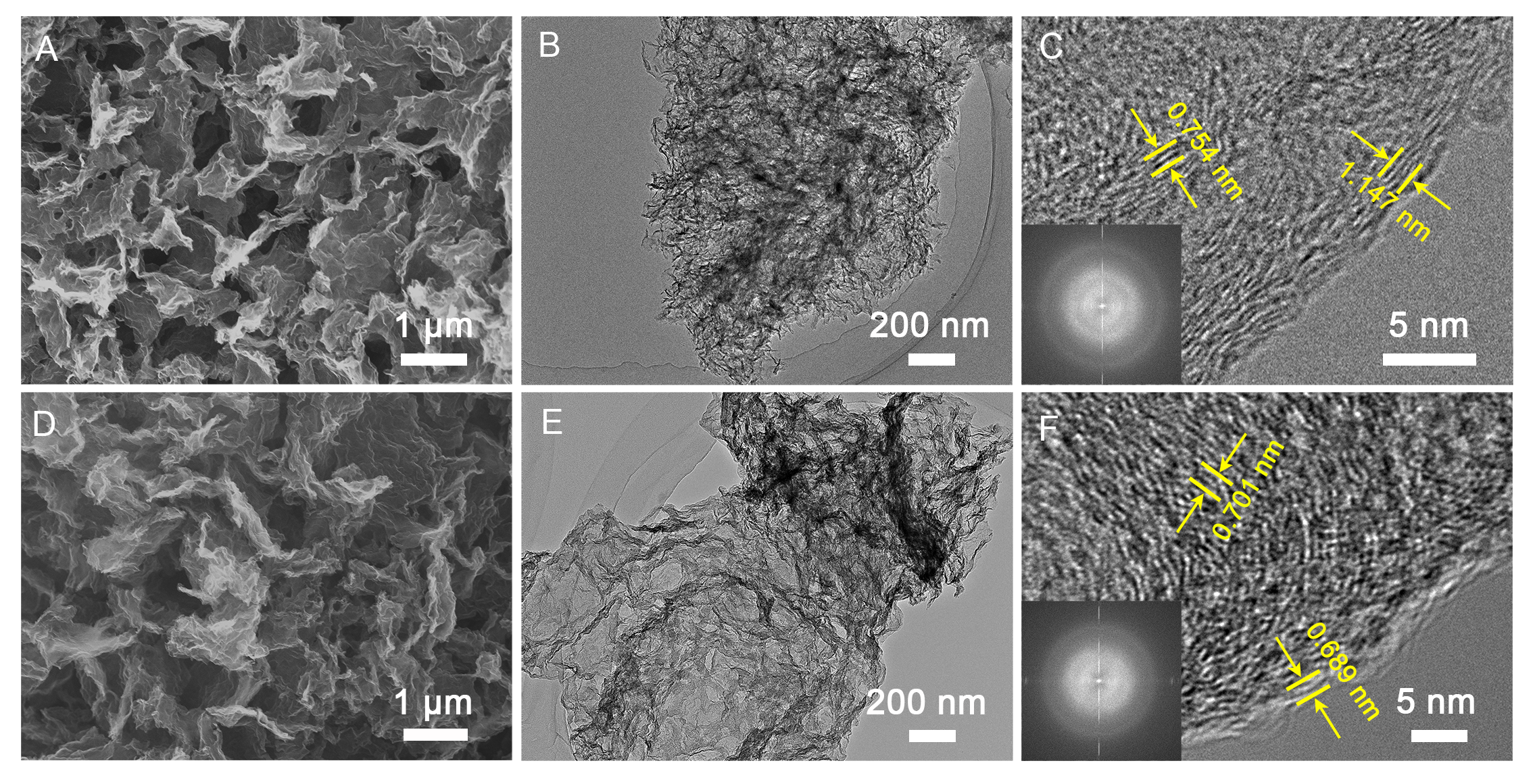


**Fig. S2.** (A) SEM, (B) TEM, and (C) HRTEM along with the FFT image of NPSC-900. (D) SEM, (E) TEM, and (F) HRTEM along with the FFT image of NPSC-1000.





**Fig. S3.** The calculation of R value.

R measured as the ratio of the height to the background of (002) peak. The background (A) is measured by drawing a straight line connecting the data on either side of the peak. The peak height (B) is determined by drawing a line tangent to the estimated linear background, which intersects the (002) peak in a single point.^4^ The R value represents the numbers of carbon sheet arranged as single layer in c direction, a smaller R value means the carbon more disordered.


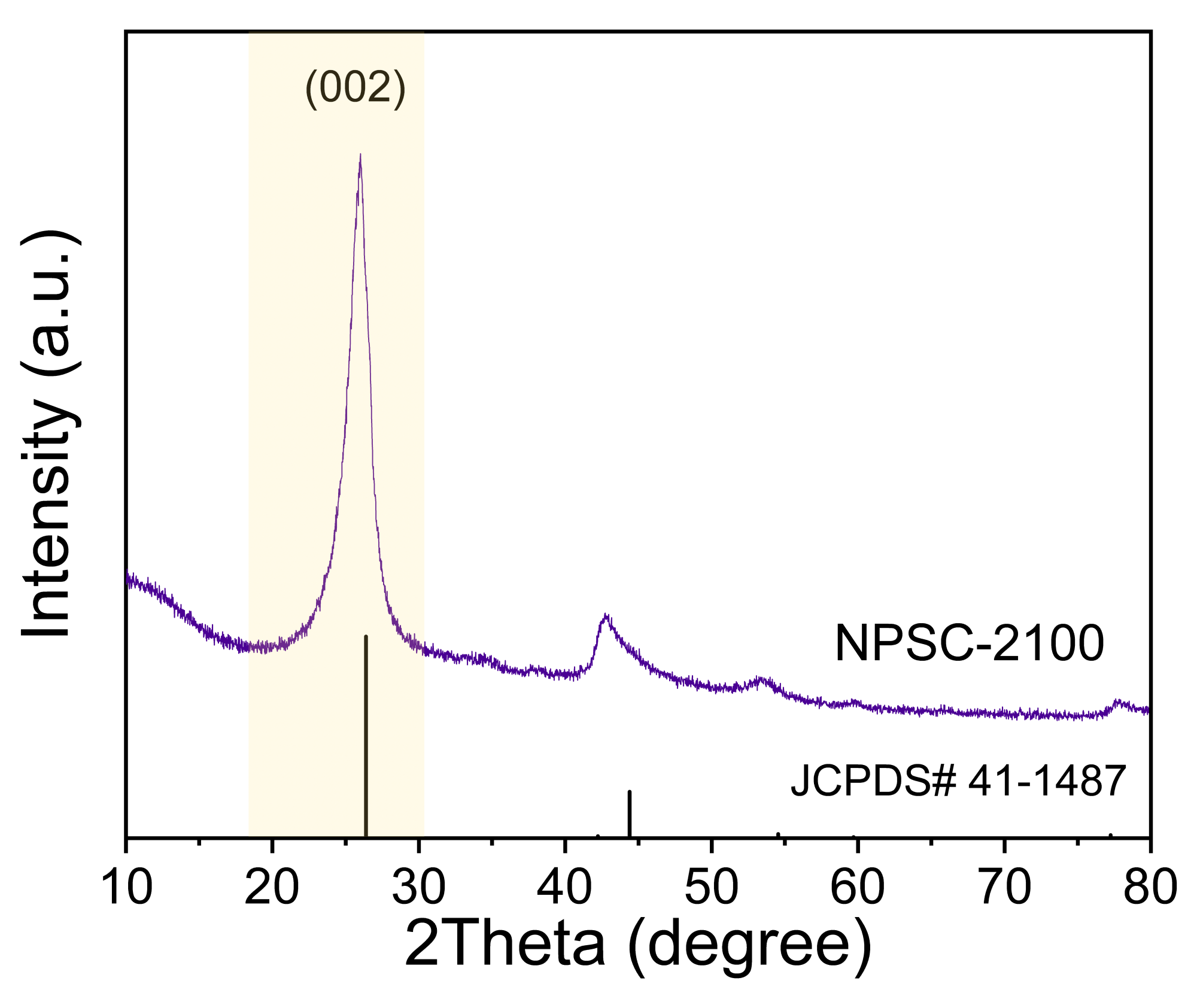


**Fig. S4.** XRD pattern of NPSC-2100.


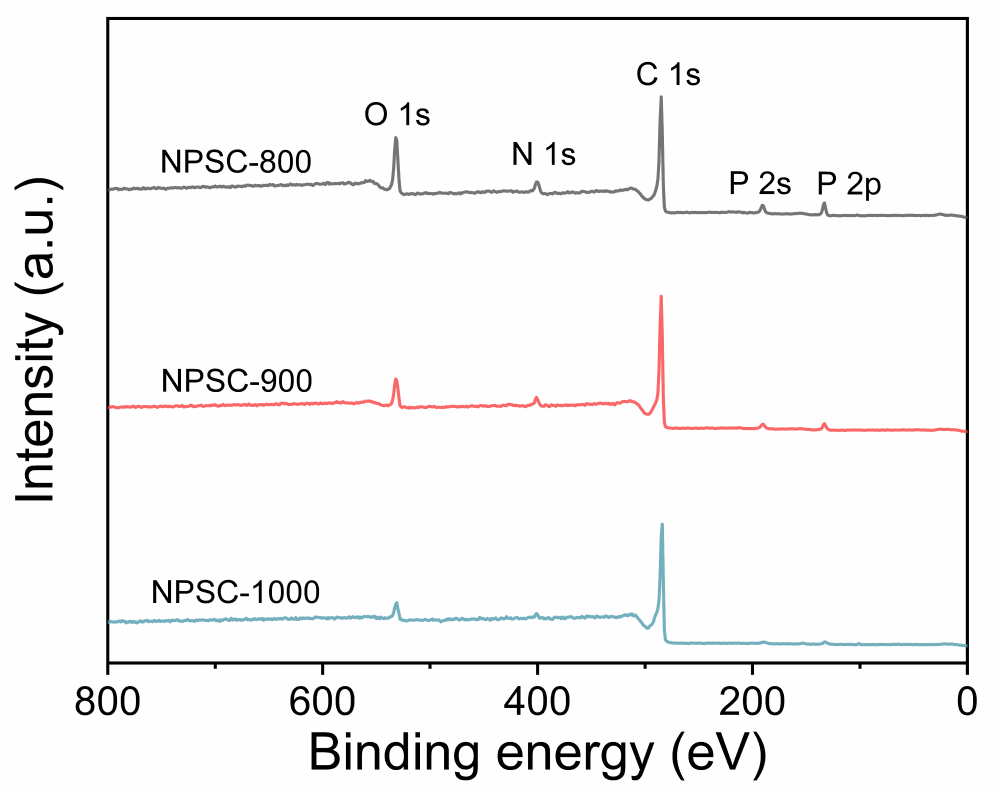


**Fig. S5.** XPS survey spectra of NPSC-800, NPSC-900, and NPSC-1000.





**Fig. S6.** Ratios of sp^3^ C to sp^2^ C (sp^3^/sp^2^) for NPSC-800, NPSC-900, and NPSC-1000.





**Fig. S7.** Ratios of N species for NPSC-800, NPSC-900, and NPSC-1000.





**Fig. S8.** Ratios of P species for NPSC-800, NPSC-900, and NPSC-1000.


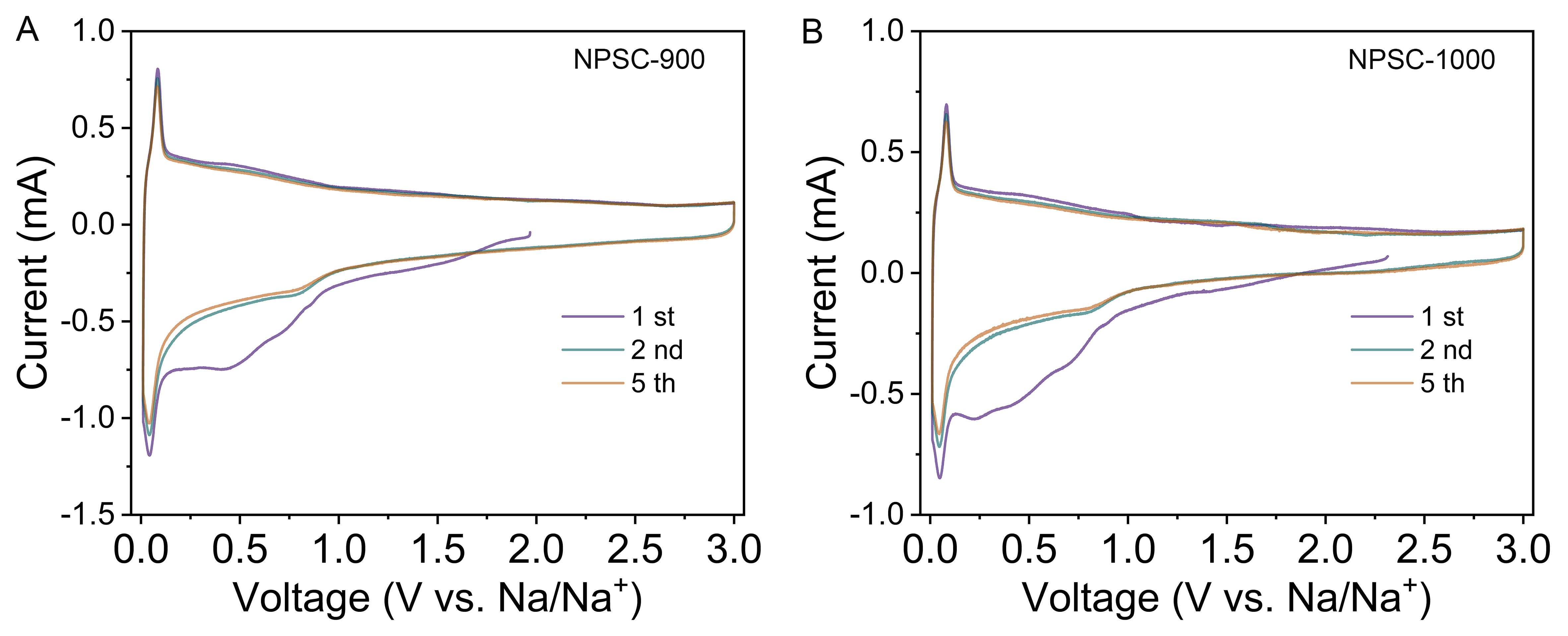


**Fig. S9.** CV curves of (A) NPSC-900 and (B) NPSC-1000 at 0.5 mV s^–1^.


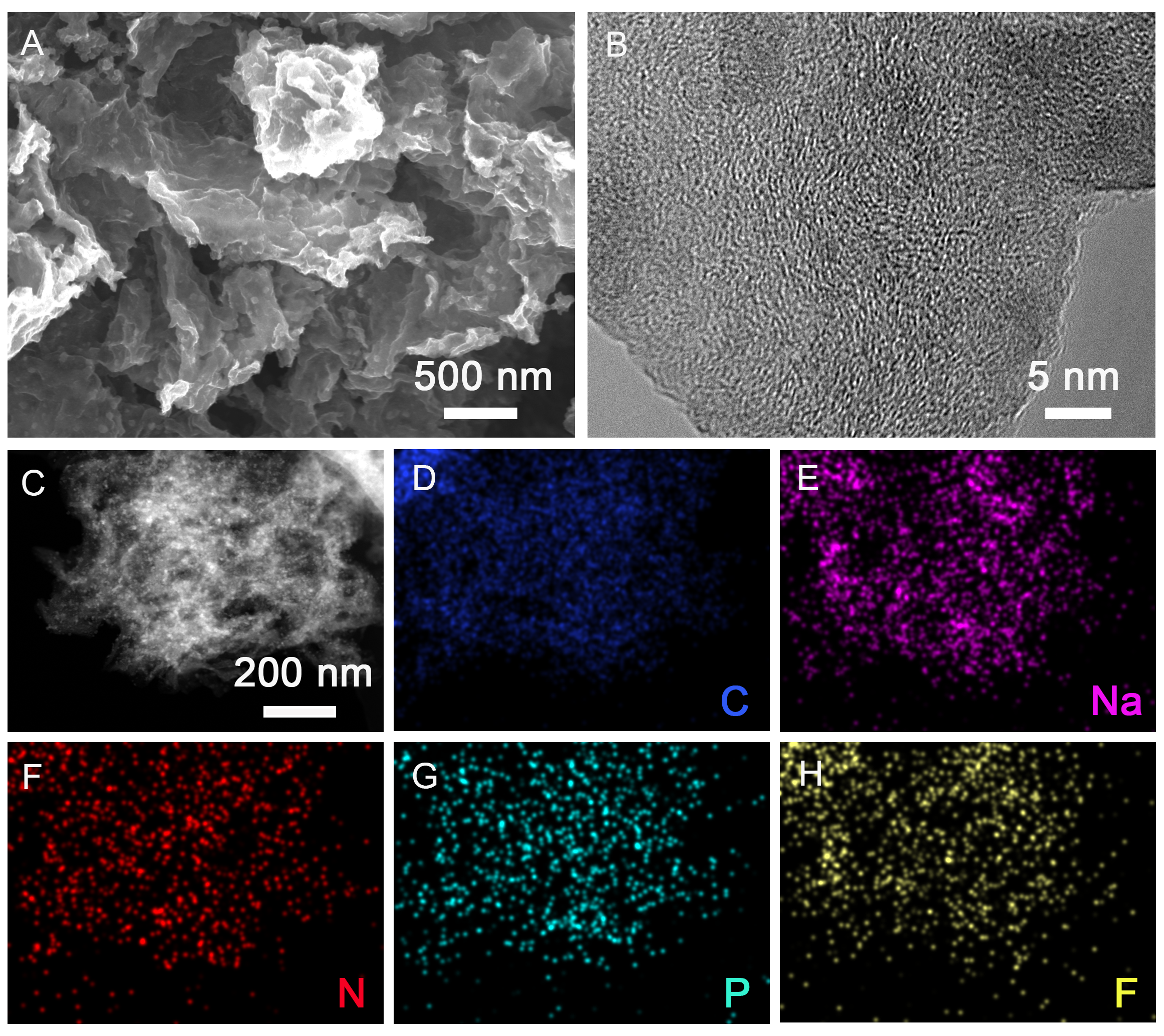


**Fig. S10.** (A) SEM, (B) HRTEM, and (C-H) the corresponding EDS mappings of NPSC-800 electrode after rate cycling.


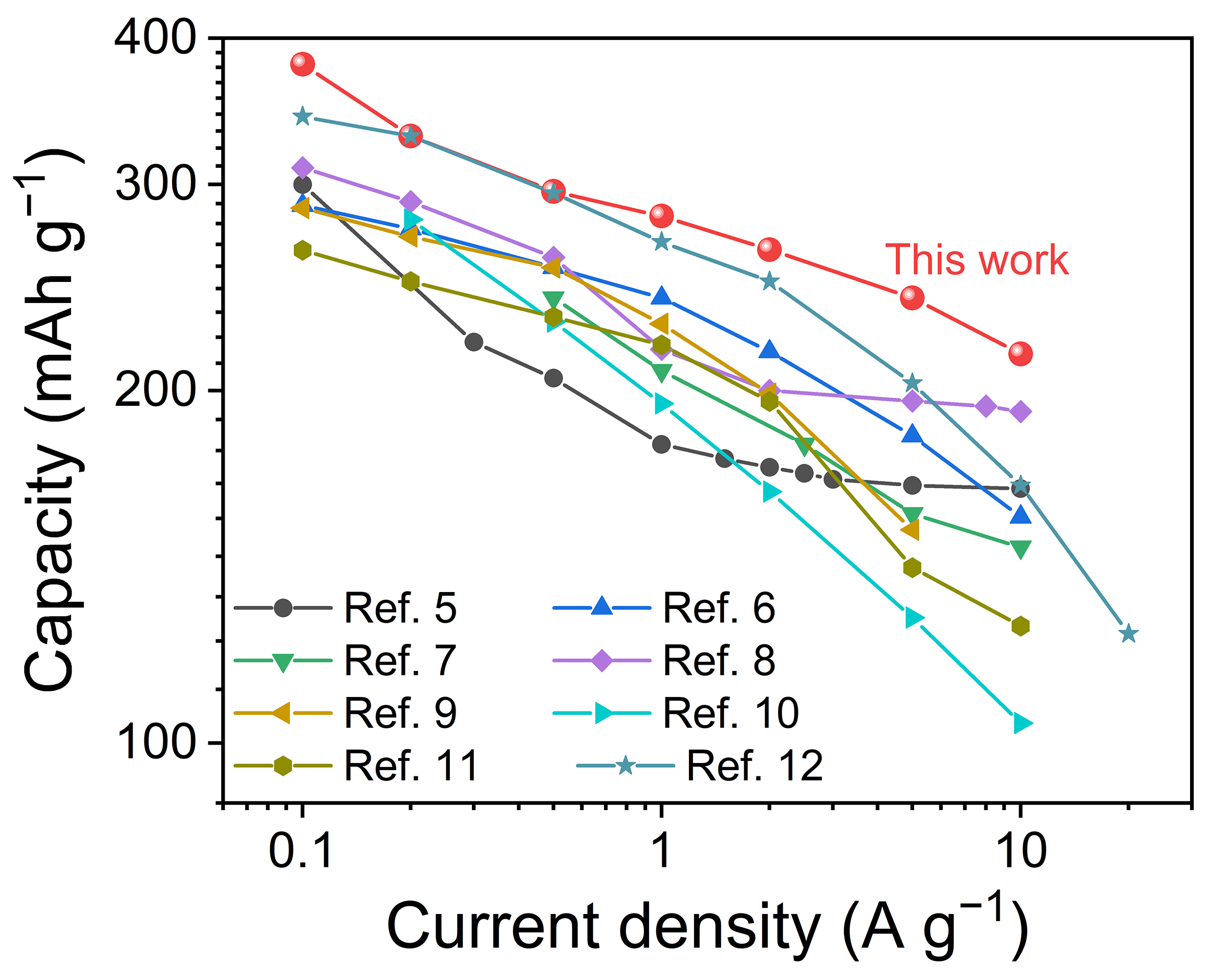


**Fig. S11.** Comparison of the rate performance of NPSC-800 with previously reported carbon materials.^5-12^





**Fig. S12.** GCD profiles of NPSC-800 at 10 A g^–1^.





**Fig. S13.** Nyquist plots of the NPSC-800, NPSC-900, and NPSC-1000 electrodes.


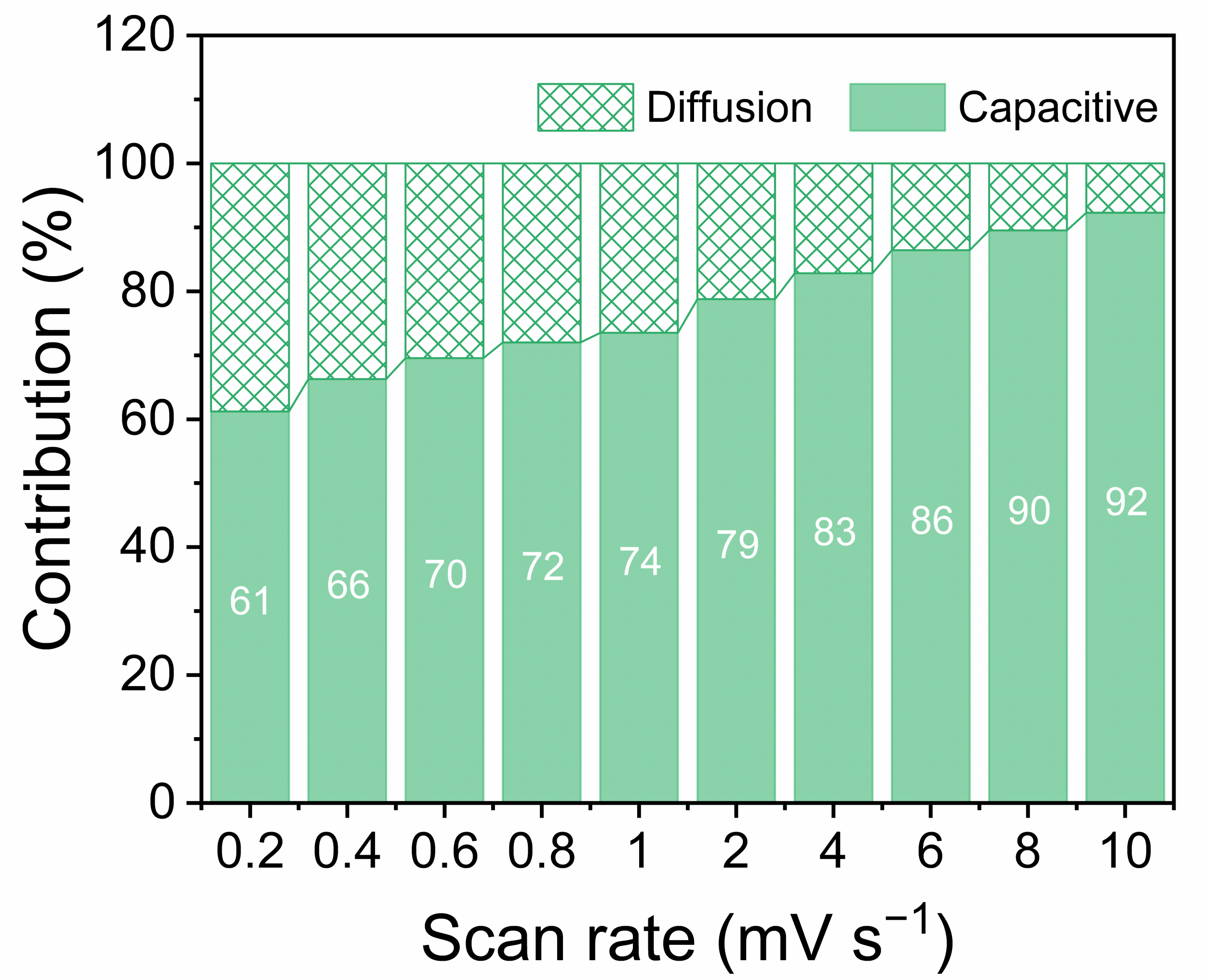


**Fig. S14.** Normalized contributions of capacitive and diffusion-controlled processes to the total capacity of NPSC-800 at various scan rates.

**
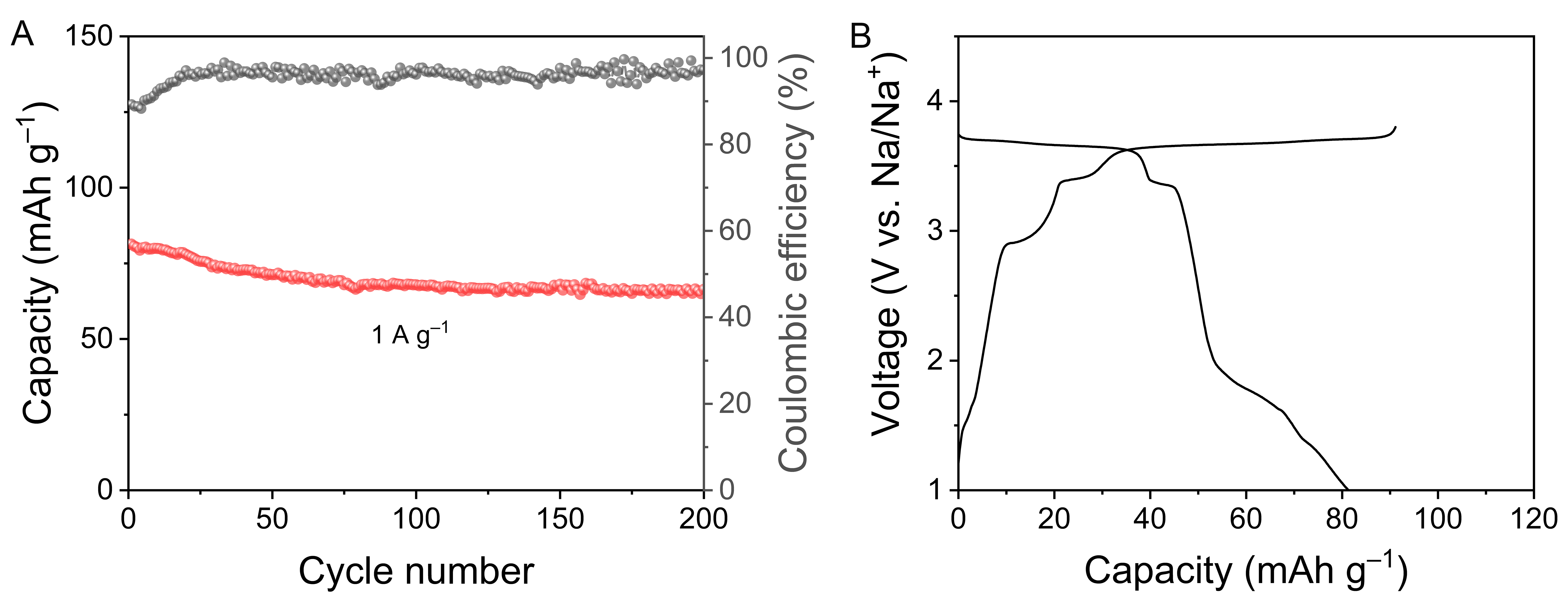
**

**Fig. S15.** (A) Cycling performance and (B) GCD profile of NVPFC at 1 A g^–1^.





**Fig. S16.** GCD profiles of the full cell at different current densities.

**Table S1.** BET analysis of NPSC-800, NPSC-900, and NPSC-1000.

| Samples | Monolayer adsorption  Volume (cm^3^ STP g^–1^) | BET surface area  (m^2^ g^–1^) | Total pore volume  (cm^3^ g^–1^) | Mesopores volume  (cm^3^ g^–1^) |
| --- | --- | --- | --- | --- |
| NPSC-800 | 51.053 | 222 | 0.8660 | 0.8288 |
| NPSC-900 | 91.123 | 397 | 1.2829 | 1.2301 |
| NPSC-1000 | 113.05 | 492 | 1.7146 | 1.6578 |

**Table S2.** XPS results of NPSC-800, NPSC-900, and NPSC-1000.

| Samples | XPS (at %) | | | |
| --- | --- | --- | --- | --- |
|  | C | N | P | O |
| NPSC-800 | 71.85 | 6.72 | 5.13 | 16.3 |
| NPSC-900 | 83.39 | 4.40 | 3.18 | 9.03 |
| NPSC-1000 | 91.53 | 1.29 | 1.73 | 5.45 |

**Table S3.** The content and percentage of different nitrogen species for NPSC-800, NPSC-900, and NPSC-1000.

| Sample | Total N (at%) | Percentage (%) | | |
| --- | --- | --- | --- | --- |
|  |  | N-Q | N-5 | N-6 |
| NPSC-800 | 6.72 | 21.0 | 58.8 | 20.2 |
| NPSC-900 | 4.40 | 24.4 | 62.8 | 12.8 |
| NPSC-1000 | 1.29 | 45.9 | 48.5 | 5.6 |

**Table S4.** The content and percentage of different phosphorus functional group for NPSC-800, NPSC-900, and NPSC-1000.

| Sample | Total P (at%) | Percentage (%) | | | |
| --- | --- | --- | --- | --- | --- |
|  |  | P-P | P-O | P-N | P-C |
| NPSC-800 | 5.13 | 0 | 9.8 | 65.7 | 24.5 |
| NPSC-900 | 3.18 | 5.4 | 10.3 | 50.4 | 33.9 |
| NPSC-1000 | 1.73 | 11.8 | 9.9 | 37.8 | 40.5 |

**Table S5.** The fitted parameters of Nyquist plots for NPSC-800, NPSC-900, and NPSC-1000.

| Sample | R_s_ (Ω) | R_ct_ (Ω) |
| --- | --- | --- |
| NPSC-800 | 5.2 | 5.2 |
| NPSC-900 | 5.3 | 15.7 |
| NPSC-1000 | 8.1 | 36.5 |

**Table S6.** Comparison of the electrochemical performance of NPSC-800 with the previously reported carbon-based anodes in SIBs.

| Samples | Voltage window  (V vs. Na/Na^+^) | Rate capacity | Cyclic stability | ICE (%) | Ref. |
| --- | --- | --- | --- | --- | --- |
| **NPSC-800** | **0.01-3.0** | **215 mAh g^–1^ at 10 A g^–1^** | **244 mAh g^–1^ at 5 A g^–1^**  **(4700 cycles)** | **81.2** | **This work** |
| N, B co-doped carbon | 0.01-3.0 | 192 mAh g^–1^ at 10 A g^–1^ | 225 mAh g^–1^ at 1 A g^–1^  (2000 cycles) | - | ^7^ |
| N, P co-doped carbon | 0.01-3.0 | 104 mAh g^–1^ at 10 A g^–1^ | 132 mAh g^–1^ at 5 A g^–1^  (2000 cycles) | 16 | ^10^ |
| S, Se, N co-doped hard carbon | 0.01-3.0 | 125.9 mAh g^–1^ at 10 A g^–1^ | 210.4 mAh g^–1^ at 1 A g^–1^  (1000 cycles) | - | ^11^ |
| MPC-1000 | 0.01-2.0 | 145 mAh g^–1^ at 5 A g^–1^ | 70 mAh g^–1^ at 5 A g^–1^  (5000 cycles) | 71 | ^13^ |
| S-doped N-rich carbon | 0.01-3.0 | 110 mAh g^–1^ at 10 A g^–1^ | 211 mAh g^–1^ at 1 A g^–1^  (1000 cycles) | 43.9 | ^14^ |
| C1600-M | 0.01-3.0 | 152 mAh g^–1^ at 2 A g^–1^ | 141 mAh g^–1^ at 1.5 A g^–1^  (2000 cycles) | 71.4 | ^15^ |
| O, N co-doped holey graphene | 0.01-3.0 | 189 mAh g^–1^ at 10 A g^–1^ | 179 mAh g^–1^ at 5 A g^–1^  (2000 cycles) | 44.1 | ^16^ |
| N, P co-doped mesoporous hard carbon | 0.01-3.0 | 150 mAh g^–1^ at 5 A g^–1^ | 197 mAh g^–1^ at 2 A g^–1^  (2000 cycles) | 40.45 | ^17^ |
| S doped disordered carbon | 0.01-3.0 | 158 mAh g^–1^ at 4 A g^–1^ | 271 mAh g^–1^ at 1 A g^–1^  (1000 cycles) | 63.2 | ^18^ |
| N, S co-doped carbon particles | 0.01-3.0 | 102 mAh g^–1^ at 10 A g^–1^ | 223 mAh g^–1^ at 1 A g^–1^  (1000 cycles) | - | ^19^ |
| SC-900 | 0.01-2.0 | 114 mAh g^–1^ at 1 A g^–1^ | 120 mAh g^–1^ at 1 A g^–1^  (240 cycles) | 67.6 | ^20^ |
| Soft carbon nanosheets | 0.01-3.0 | 103.8 mAh g^–1^ at 1 A g^–1^ | 128.7 mAh g^–1^ at 0.8 A g^–1^  (3500 cycles) | - | ^21^ |
| Mesoporous soft carbon | 0.01-3.0 | 53 mAh g^–1^ at 10 A g^–1^ | 103 mAh g^–1^ at 0.5 A g^–1^  (3000 cycles) | 45 | ^22^ |

**2. Supplementary References**

1. Lu, X.; Li, L.; Song, B.; Moon, K.-s.; Hu, N.; Liao, G.; Shi, T.; Wong, C., Mechanistic investigation of the graphene functionalization using p-phenylenediamine and its application for supercapacitors. *Nano Energy* **2015,** *17*, 160.

2. Shan, Z.; Wu, M.; Zhu, D.; Wu, X.; Zhang, K.; Verduzco, R.; Zhang, G., 3D Covalent Organic Frameworks with Interpenetrated pcb Topology Based on 8-Connected Cubic Nodes. *J. Am. Chem. Soc.* **2022,** *144* (13), 5728.

3. Ai, W.; Wang, X.; Zou, C.; Du, Z.; Fan, Z.; Zhang, H.; Chen, P.; Yu, T.; Huang, W., Molecular-Level Design of Hierarchically Porous Carbons Codoped with Nitrogen and Phosphorus Capable of In Situ Self-Activation for Sustainable Energy Systems. *Small* **2017,** *13* (8), 1602010.

4. Qi, Y.; Lu, Y.; Ding, F.; Zhang, Q.; Li, H.; Huang, X.; Chen, L.; Hu, Y. S., Slope-Dominated Carbon Anode with High Specific Capacity and Superior Rate Capability for High Safety Na-Ion Batteries. *Angew. Chem. Int. Ed.* **2019,** *58* (13), 4361.

5. Guo, R.; Lv, C.; Xu, W.; Sun, J.; Zhu, Y.; Yang, X.; Li, J.; Sun, J.; Zhang, L.; Yang, D., Effect of Intrinsic Defects of Carbon Materials on the Sodium Storage Performance. *Adv. Energy Mater.* **2020,** *10* (9), 1903652.

6. Ni, D.; Sun, W.; Wang, Z.; Bai, Y.; Lei, H.; Lai, X.; Sun, K., Heteroatom‐Doped Mesoporous Hollow Carbon Spheres for Fast Sodium Storage with an Ultralong Cycle Life. *Adv. Energy Mater.* **2019,** *9* (19), 1900036.

7. Jin, Q.; Li, W.; Wang, K.; Li, H.; Feng, P.; Zhang, Z.; Wang, W.; Jiang, K., Tailoring 2D Heteroatom‐Doped Carbon Nanosheets with Dominated Pseudocapacitive Behaviors Enabling Fast and High‐Performance Sodium Storage. *Adv. Funct. Mater.* **2020,** *30* (14), 1909907.

8. Wang, G.; Shao, M.; Ding, H.; Qi, Y.; Lian, J.; Li, S.; Qiu, J.; Li, H.; Huo, F., Multiple Active Sites of Carbon for High-Rate Surface-Capacitive Sodium-Ion Storage. *Angew. Chem. Int. Ed.* **2019,** *58* (38), 13584.

9. Li, N.; Yang, Q.; Wei, Y.; Rao, R.; Wang, Y.; Sha, M.; Ma, X.; Wang, L.; Qian, Y., Phosphorus-doped hard carbon with controlled active groups and microstructure for high-performance sodium-ion batteries. *J. Mater. Chem. A* **2020,** *8* (39), 20486.

10. Liu, H.; Zeng, W.; Yang, Y.; Chen, J.; Zhao, Y.; Mu, S., Synchronously improved graphitization and surface area in a 3D porous carbon network as a high capacity anode material for lithium/sodium-ion batteries. *J. Mater. Chem. A* **2021,** *9* (2), 1260.

11. Liu, Y.; Dai, H.; An, Y.; Fu, L.; An, Q.; Wu, Y., Facile and scalable synthesis of a sulfur, selenium and nitrogen co-doped hard carbon anode for high performance Na- and K-ion batteries. *J. Mater. Chem. A* **2020,** *8* (30), 14993.

12. Yan, R.; Leus, K.; Hofmann, J. P.; Antonietti, M.; Oschatz, M., Porous nitrogen-doped carbon/carbon nanocomposite electrodes enable sodium ion capacitors with high capacity and rate capability. *Nano Energy* **2020,** *67*, 104240.

13. Xia, J. L.; Yan, D.; Guo, L. P.; Dong, X. L.; Li, W. C.; Lu, A. H., Hard Carbon Nanosheets with Uniform Ultramicropores and Accessible Functional Groups Showing High Realistic Capacity and Superior Rate Performance for Sodium-Ion Storage. *Adv. Mater.* **2020,** *32* (21), 2000447.

14. Yang, J.; Zhou, X.; Wu, D.; Zhao, X.; Zhou, Z., S-Doped N-Rich Carbon Nanosheets with Expanded Interlayer Distance as Anode Materials for Sodium-Ion Batteries. *Adv. Mater.* **2017,** *29* (6), 1604108.

15. Sun, F.; Wang, H.; Qu, Z.; Wang, K.; Wang, L.; Gao, J.; Gao, J.; Liu, S.; Lu, Y., Carboxyl‐Dominant Oxygen Rich Carbon for Improved Sodium Ion Storage: Synergistic Enhancement of Adsorption and Intercalation Mechanisms. *Adv. Energy Mater.* **2020,** *11* (1), 2002981.

16. Zhao, J.; Zhang, Y. Z.; Chen, J.; Zhang, W.; Yuan, D.; Chua, R.; Alshareef, H. N.; Ma, Y., Codoped Holey Graphene Aerogel by Selective Etching for High‐Performance Sodium‐Ion Storage. *Adv. Energy Mater.* **2020,** *10* (18), 2000099.

17. Tao, S.; Xu, W.; Zheng, J.; Kong, F.; Cui, P.; Wu, D.; Qian, B.; Chen, S.; Song, L., Soybean roots-derived N, P Co-doped mesoporous hard carbon for boosting sodium and potassium-ion batteries. *Carbon* **2021,** *178*, 233.

18. Li, W.; Zhou, M.; Li, H.; Wang, K.; Cheng, S.; Jiang, K., A high performance sulfur-doped disordered carbon anode for sodium ion batteries. *Energy Environ. Sci.* **2015,** *8* (10), 2916.

19. Jin, Q.; Wang, K.; Feng, P.; Zhang, Z.; Cheng, S.; Jiang, K., Surface-dominated storage of heteroatoms-doping hard carbon for sodium-ion batteries. *Energy Storage Mater.* **2020,** *27*, 43.

20. Luo, W.; Jian, Z.; Xing, Z.; Wang, W.; Bommier, C.; Lerner, M. M.; Ji, X., Electrochemically Expandable Soft Carbon as Anodes for Na-Ion Batteries. *ACS Cent. Sci.* **2015,** *1* (9), 516.

21. Yao, X.; Ke, Y.; Ren, W.; Wang, X.; Xiong, F.; Yang, W.; Qin, M.; Li, Q.; Mai, L., Defect‐Rich Soft Carbon Porous Nanosheets for Fast and High‐Capacity Sodium‐Ion Storage. *Adv. Energy Mater.* **2018,** *9* (6), 1803260.

22. Cao, B.; Liu, H.; Xu, B.; Lei, Y.; Chen, X.; Song, H., Mesoporous soft carbon as an anode material for sodium ion batteries with superior rate and cycling performance. *J. Mater. Chem. A* **2016,** *4* (17), 6472.
